# Effects of short-term glucocorticoid treatment on changes in cartilage matrix degradation and chondrocyte gene expression induced by mechanical injury and inflammatory cytokines

**DOI:** 10.1186/ar3456

**Published:** 2011-09-02

**Authors:** Yihong CS Lu, Christopher H Evans, Alan J Grodzinsky

**Affiliations:** 1 Department of Biological Engineering, MIT, 500 Technology Square NE47-377, Cambridge, MA, 02139, USA; 2 Department of Orthopaedic Surgery, Beth Israel Deaconess Medical Center, 330 Brookline Avenue, RN-115, Boston, MA, 02215 USA; 3 Departments of Electrical Engineering and Computer Science and Mechanical Engineering, MIT, 500 Technology Square NE47-377, Cambridge, MA, 02139, USA

## Abstract

**Introduction:**

Traumatic joint injury damages cartilage and causes adjacent joint tissues to release inflammatory cytokines, increasing the risk of developing osteoarthritis. The main objective of this study was to determine whether the combined catabolic effects of mechanical injury, tumor necrosis factor alpha (TNFα) and interleukin-6 (IL-6)/soluble IL-6 receptor (sIL-6R) on cartilage could be abolished by short-term treatment with glucocorticoids such as dexamethasone.

**Methods:**

In an initial dexamethasone-dose-response study, bovine cartilage explants were treated with TNFα and increasing concentrations of dexamethasone. Bovine and human cartilage explants were then subjected to individual and combined treatments with TNFα, IL-6/sIL-6R and injury in the presence or absence of dexamethasone. Treatment effects were assessed by measuring glycosaminoglycans (GAG) release to the medium and synthesis of proteoglycans. Additional experiments tested whether pre-exposure of cartilage to dexamethasone could prevent GAG loss and inhibition of biosynthesis induced by cytokines, and whether post-treatment with dexamethasone could diminish the effects of pre-established cytokine insult. Messenger ribonucleic acid (mRNA) levels for genes involved in cartilage homeostasis (proteases, matrix molecules, cytokines, growth and transcription factors) were measured in explants subjected to combined treatments with injury, TNFα and dexamethasone. To investigate mechanisms associated with dexamethasone regulation of chondrocyte metabolic response, glucocorticoid receptor (GR) antagonist (RU486) and proprotein convertase inhibitor (RVKR-CMK) were used.

**Results:**

Dexamethasone dose-dependently inhibited GAG loss and the reduction in biosynthesis caused by TNFα. The combination of mechanical injury, TNFα and IL-6/sIL-6R caused the most severe GAG loss; dexamethasone reduced this GAG loss to control levels in bovine and human cartilage. Additionally, dexamethasone pre-treatment or post-treatment of bovine explants lowered GAG loss and increased proteoglycan synthesis in cartilage explants exposed to TNFα. Dexamethasone did not down-regulate aggrecanase mRNA levels. Post-transcriptional regulation by dexamethasone of other genes associated with responses to injury and cytokines was noted. GR antagonist reversed the effect of dexamethasone on sulfate incorporation. RVKR-CMK significantly reduced GAG loss caused by TNFα + IL-6 + injury.

**Conclusions:**

Short-term glucocorticoid treatment effectively abolished the catabolic effects exerted by the combination of pro-inflammatory cytokines and mechanical injury: dexamethasone prevented proteoglycan degradation and restored biosynthesis. Dexamethasone appears to regulate the catabolic response of chondrocytes post-transcriptionally, since the abundance of transcripts encoding aggrecanases was still elevated in the presence of dexamethasone.

## Introduction

Osteoarthritis (OA) is characterized by chronic, irreversible degradation of articular cartilage. Traumatic joint injury in young adults greatly increases the risk of developing OA [1,2] and post-traumatic OA remains a major clinical and societal problem. Treatments following joint trauma initially focus on reducing pain and swelling, and often by subsequent reconstructive surgery to stabilize joint biomechanics, for example, for injuries involving anterior cruciate ligament (ACL) rupture. However, these interventions do not prevent the progression to secondary OA after injury [3,4]. Following knee injury, high levels of aggrecan fragments and cross-linked peptides from type II collagen accumulate in the synovial fluid [5]. Moreover, joint injury results in an immediate surge in synovial fluid concentrations of pro-inflammatory cytokines, including tumor necrosis factor-α (TNFα), interleukin-1β (IL-1β), IL-6 and IL-8 [6-8]. The levels of these cytokines remain elevated for weeks and eventually decrease to levels detected in chronic OA joints [8]. Thus, cartilage in the injured joint is often subjected to an initial biomechanical insult [9] and then further compromised by the presence of high levels of inflammatory cytokines [10].

In a recent report, we highlighted the interplay between mechanical and cytokine-mediated pathways regulating cartilage degradation relevant to traumatic joint injury [11]. We used an *in vitro *model involving injurious compression of cartilage explants to simulate the initial mechanical insult, and subsequent co-culture with exogenous cytokines to simulate the inflammatory component. In both human and bovine cartilage, mechanical injury and TNFα synergistically increased proteoglycan degradation [11]. Moreover, mechanical injury potentiated the combined catabolic effects of TNFα and IL-6 along with its soluble receptor, sIL-6R, causing the most severe glycosaminoglycan (GAG) loss among all treatment conditions. Proteoglycan degradation was found to be mediated by aggrecanase activity [11] in these studies.

In the present study, we address the potential utility of glucocorticoids (GCs) in the treatment of joint injury. Intra-articular injection of GCs is an established treatment for both chronic OA and rheumatoid arthritis (RA) [12,13]. GCs exert their effects by binding to intracellular glucocorticoid receptors (GRs), which act as transcription factors in cells. The activated GRs either directly or indirectly regulate the transcription of target genes. For example, GRs are known to enhance the production of anti-inflammatory cytokines such as IL-1 receptor antagonist and IL-10 [14], while the expression of molecules associated with inflammatory processes, including cytokines IL-1β, IL-6, TNFα, and cyclooxygenase-2 [15-18] is repressed. The effects of GCs in cartilage are less well understood. Since human chondrocytes have been shown to express GRs [19,20], the potential effects of GCs in treating joint disorders may be due to direct regulation of chondrocytes, but this possibility has not been widely studied.

Dexamethasone (DEX) is a very potent synthetic GC due to its high receptor binding affinity [21]. DEX has been commonly used in cartilage tissue engineering; numerous studies have demonstrated that DEX potentiates the ability of progenitor cells to undergo chondrogenic differentiation and to synthesize cartilage proteoglycans [22-24]. However, the effects of DEX on cartilage matrix turnover, particularly those changes associated with joint injury, remain unclear.

The objectives of this study were: (1) to test the hypothesis that short-term treatment with DEX could abolish matrix degradation and the known reduction of chondrocyte biosynthesis caused by the combination of mechanical injury and inflammatory cytokines in bovine and human cartilage explants, (2) to investigate whether DEX regulates this metabolic response at the transcriptional level in chondrocytes, and (3) to explore mechanistic pathways by which DEX may suppress cartilage degradation. The pathways of interest included regulation of aggrecanase gene expression and the activation of aggrecanases by proprotein convertases, the effects of DEX on inducible nitric oxide synthase (iNOS) mRNA and protein levels, and the role of glucocorticoid receptors.

A disintegrin and metalloproteinase with thrombospondin motifs-4,-5 (ADAMTS-4 and -5) are the primary aggrecanases responsible for the pathological process of aggrecan degradation in human OA [25]. Aggrecanases are synthesized as latent, inactive enzymes whose pro-domains must be removed by proprotein convertases (PCs) in order to express their catalytic function. Studies have shown increased activity of PCs in both osteoarthritic and cytokine-stimulated cartilage, and inhibiting PC activity significantly reduced cytokine-induced aggrecan degradation [26]. Among the PCs, furin, PACE4 and PC5/6 are capable of removing the prodomain of ADAMTS-4 [27], while furin and PC7 have been shown to process pro-ADAMTS-5 [28]. Thus, regulation of aggrecanase activation as well as mRNA levels of ADAMTS-4 and -5 are both pathways of interest.

## Materials and methods

After a description of cartilage explant harvest and the methods for applying injurious mechanical compression to these explants, we then delineated methods to test the effects DEX on matrix metabolism in explants subjected to mechanical injury and inflammatory cytokine challenge. In one series of experiments using bovine and human cartilage, DEX was added immediately at the time of injury and cytokine treatment. In another series of experiments using bovine tissue, DEX was added either two days before or two days after injury + cytokine treatment to test whether DEX could protect and/or rescue changes in cartilage matrix metabolism caused by injury. The concentration of DEX used in all these tests was determined from an initial dose-response study. We then describe methods for experiments focusing on mechanistic pathways, including studies of DEX regulation of chondrocyte transcription, effects of DEX on iNOS mRNA and protein levels, and inhibition of glucocorticoid receptors and proprotein convertases.

### Bovine cartilage harvest and culture

Cartilage disks were harvested from the femoropatellar grooves of one- to two-week-old bovine calf knee joints (obtained from Research 87, Hopkinton, MA, USA) as previously described [29]. A total of 16 joints from 13 different animals and 1 human were used. Briefly, cartilage-bone cylinders (9 mm diameter) were cored perpendicular to the surface. After a level surface was obtained by removing the most superficial layer (approximately 100 to 200 μm), one to two sequential 1 mm-slices of middle zone cartilage were cut from each cylinder. Five disks (3 mm-diameter, 1 mm-thick) were cored from each slice using a dermal punch. Cartilage from this middle zone in newborn calves was shown previously to have a reasonably homogeneous population of cells and matrix [30]. Cartilage disks for all treatment groups were matched for depth and location along the joint surface [31]. Disks were equilibrated in serum-free medium (low-glucose DMEM (Cellgro, Herndon, VA, USA)), 10 mM HEPES buffer (Invitrogen, Carlsbad, CA, USA), supplemented with 1% insulin-transferrin-selenium (10 μg/ml, 5.5 μg/ml and 5 ng/ml, respectively), 0.1 mM nonessential amino acids, 0.4 mM proline, 20 μg/mL ascorbic acid, 100 units/mL penicillin G, 100 μg/mL streptomycin, and 0.25 μg/mL amphotericin B (all from Sigma, St. Louis, MO, USA)) for two days prior to treatment in a 37°C, 5% CO_2 _incubator.

### Postmortem adult human donor tissue

Human donor knee cartilage (49-yr-old female, modified-Collins [32] grade-1 knee joint) was obtained from the Gift of Hope Organ and Tissue Donor Network (Elmhurst, IL, USA), approved by the Office of Research Affairs at Rush-Presbyterian-St. Luke's Medical Center and the Committee on Use of Humans as Experimental Subjects at MIT. Any fibrillated areas detected under visual inspection were excluded from the study. Human cartilage harvest and culture were identical to that of bovine, but included the intact superficial zone and each disk was approximately 0.8 mm thick. Human knee cartilage was obtained from both the femoropatellar groove and femoral condyles.

### Injurious compression

After equilibration in medium for three days, disks were injuriously compressed in a custom-designed incubator-housed apparatus [33,34]. Each bovine disk was subjected to radially unconfined compression to 50% final strain at 1 mm/second velocity (100% per second strain rate), followed by immediate release of load at the same rate, as described [29]. Immediately after injury, some disks were deformed to an ellipsoidal shape (deformation score of 1 or 2 as described in [35]), but none exhibited gross fissuring. Adult human cartilage disks were thinner, had intact superficial zone and different effective biomechanical behavior compared to the immature bovine disks, reflecting the anisotropy and inhomogeneity associated with the presence of the superficial zone. The combined properties were such that higher strain and strain rate values were needed to produce levels of peak stress and visible deformation in human cartilage similar to that observed for immature bovine tissue [29]. Thus, a strain of 60% and strain rate of 300%/second were used, the same values utilized in our recent report with this *in vitro *injury plus cytokine stimulation system for adult human cartilage [11]. The resulting macroscopic tissue changes in human cartilage disks were similar (elliptical appearance) to those described previously using our human cartilage injury model and scoring system [36]. After injury, samples were immediately placed in treatment medium.

### DEX dose-response

In a DEX dose-response study, bovine cartilage samples (70 disks from two joints of one animal) were treated either with or without rhTNFα (25 ng/mL) and incubated for six days with increasing concentrations of DEX (Sigma, St. Louis, MO, USA), from 0.1 nM to 100 μM.

### Exogenous cytokines, injury and DEX treatments

Cartilage samples were either subjected to injurious compression or left uninjured, incubated with or without cytokines (all from R&D Systems, Minneapolis, MN, USA), and with or without DEX. Previously [11], we observed that treatments with TNFα, TNFα + injury, TNFα + IL-6/sIL-6R, and TNFα + injury + IL-6/sIL-6R caused significant release of GAGs from both human and bovine cartilage explants, with the latter condition causing the most severe loss of GAG. In this study, we first examined the effects of DEX on cartilage explants under these same conditions. For bovine cartilage (70 disks from two joints of another animal), DEX and recombinant human TNFα (rhTNFα) were used at 10 nM and 25 ng/mL, respectively, based on the results from the DEX dose response study. For human cartilage (36 disks from the distal femur), DEX and rhTNFα were used at 100 nM and 100 ng/mL, respectively. rhIL-6 (50 ng/mL) was always used in combination with soluble IL-6 receptor (sIL-6R, 250 ng/mL), since this combination was found previously to induce greater aggrecan degradation than when used separately in the presence of TNFα [37]. Bovine cartilage disks were cultured in these conditions for six days. Culture duration for human explants was extended to 10 days based on earlier studies showing that human cartilage released sGAG more slowly than bovine cartilage for these conditions [11]. Medium was replaced every two days and saved for analysis.

### Pre- and post-treatment with DEX

To test whether a short-duration pre-exposure of cartilage to DEX could prevent GAG loss and inhibition of biosynthesis induced by subsequent cytokine treatment, bovine cartilage disks (10 disks from a separate animal) were either pre-treated with DEX for two days or incubated in medium alone. Afterwards, both groups were incubated in medium containing TNFα but no DEX for an additional four days. To test whether post-treatment with DEX could diminish the effects of a pre-established cytokine insult, cartilage explants (10 disks from a different animal) were first treated with TNFα for two days, and DEX was then added to the medium in addition to continued treatment with TNFα for another four days. GAG loss and radiolabel incorporation were measured as above.

### Matrix biosynthesis and biochemical analyses

Two days before termination of the bovine cultures, the medium of each disk was supplemented with 5 μCi/ml Na_2_^35^SO_4 _(Perkin-Elmer, Norwalk, CT, USA) as a measure of the rate of proteoglycan synthesis. The amount of radiolabeled sulfate was doubled in studies of human cartilage. Upon termination, disks were washed, weighed and solubilized (proteinase K, Roche, Indianapolis, IN, USA), and radiolabel incorporation was measured using a liquid scintillation counter [30]. The amounts of GAG lost to the medium and retained in the cartilage were measured using the dimethylmethylene blue (DMMB) assay, with shark chondroitin sulfate (Sigma) as the standard [38].

### Gene expression studies: RNA extraction and real-time PCR

To examine the effects of DEX, injury and TNFα on chondrocyte gene expression, bovine cartilage disks from six different animals were cultured for four days under the eight treatment conditions: (1) no-treatment control, (2) DEX-only, (3) mechanical injury only, (4) DEX + injury, (5) TNFα, (6) TNFα + DEX, (7) TNFα + injury, and (8) TNFα + injury + DEX. A total of 48 disks per animal from each of six different joints (six different animals) were used. From each joint, RNA was pooled from the six disks assigned to each of the eight treatment conditions (matching disks from along the joint surface across treatment groups). Thus, there were six different repeats of this experiment in total, with each repeat corresponding to a different joint (animal). Samples were pulverized in liquid nitrogen and homogenized in TRIzol reagent (Invitrogen). The extract was spun at 13,000 g for 10 minutes in Phase Gel tubes (Eppendorf, Hamburg, Germany) with 10% chloroform (Sigma). After spinning, the clear supernatant was obtained and RNA was isolated using the RNeasy Mini columns (Qiagen, Chatsworth, CA, USA); genomic DNA was removed by a DNase digestion step (Qiagen) according to the manufacturer's protocol. Absorbance was read at 260 nm and 280 nm to measure the concentration of RNA and the purity of the extract. Reverse transcription of equal quantities of RNA (2.5 μg) from each condition was performed using the AmpliTaq-Gold Reverse Transcription Kit (Applied Biosystems, Foster City, CA, USA) [39]. Genes of interests were those involved in cartilage homeostasis, including matrix molecules (aggrecan, collagen II and IX), cytokines (IL-1β, IL-6, TNFα), proteases and protease inhibitors (ADAMTS-4,-5, matrix metalloproteinase-3 (MMP-3), tissue inhibitor of metalloproteinase-3 (TIMP-3)), iNOS and a housekeeping gene (18 S). Bovine primer sequences for all genes except iNOS, collagen IX and IL-6 were reported in our previous studies [40,41]; sequences for these latter three genes were reported in another study [42]; they were also designed using Primer3 software [43] on the basis of bovine sequences. A standard curve for amplification was generated for each of the primer. All primers demonstrated approximately equally efficiency, with standard curve slopes of approximately 1, indicating a doubling in complementary DNA quantity in each cycle [39]. Real-time PCR was performed using Applied Biosystems ABI 7900HT instrument and SYBR Green Master Mix (Applied Biosystems). Measured threshold values (Ct) were converted to RNA copy number according to primer efficiencies. Within each condition, the RNA copy numbers for each gene were normalized to that of 18 S from the same condition. To examine the effects of treatments, each gene was then normalized to its level in the no-treatment control group.

### Pathways: inhibition of glucocorticoid receptor, proprotein convertases and iNOS

The role of chondrocyte GRs in the response to DEX was studied in bovine cartilage samples (30 disks from one animal) by treatment with the GR antagonist, RU486 (1 μM, Sigma), in the presence of TNFα and TNFα + DEX for six days. The role of proprotein convertases in matrix degradation was tested by the addition of the PC inhibitor decanoyl-RVKR-CMK (10 μM, Calbiochem, La Jolla, CA, USA) to bovine cartilage explants (35 disks from one animal) cultured with different combinations of TNFα, IL-6/sIL-6R and mechanical injury. The levels of iNOS protein were measured following four-day treatments of bovine cartilage disks with TNFα ± injury, in the presence or absence of DEX. The disks were then pulverized in liquid nitrogen and homogenized in buffer solution (20 mM pH 7.6 Tris, 120 Mm NaCl, 10 mM EDTA, 10% glycerol, 1% Nonidet P-40 (Sigma) with protease inhibitor cocktail (Roche)). Equal amounts of protein were collected from each condition, run on 4 to 15% gels (Invitrogen) and then transferred to polyvinylidene difluoride (BioRad, Hercules, CA, USA) for immunoblotting. Western blots were performed using anti-bovine iNOS antibody (1:1000, Millipore, Billerica, MA, USA), followed by secondary antibodies conjugated to horseradish peroxidase (1:4000, Cell Signaling Technology, Beverly, MA, USA). In another study, nitrite levels in the medium of 48 disks (one animal) were analyzed using the Griess Reagents (Invitrogen).

### Statistical analyses

In studying the effect of DEX dose on GAG loss and proteoglycan biosynthesis, a general linear model was used to analyze the data, followed by Dunnet's test for comparisons to controls. In evaluating the effect of DEX on GAG loss, sulfate incorporation and nitrite accumulation in cytokine-treated and mechanically-injured bovine and human cartilage, as well as the effect of CMK on GAG loss in bovine cartilage, a general linear model with Bonferroni's test was used to conduct hypothesis-based comparisons. For the study testing the effect of RU486, a general linear model was used followed with Tukey's test. In the studies of pre- and post-treatment of cartilage with DEX, a two-way general linear model with Tukey's test was used to evaluate differences between conditions and time points. For gene expression studies, log-transformed expression data were analyzed using a general linear model followed by Dunnet's test for comparison of each of the conditions to no-treatment controls. All values are expressed as mean ± SEM, with *P *≤0.05 taken as statistically significant. Statistical analyses were performed using SYSTAT-12 software (Richmond, CA, USA).

## Results

### DEX dose-dependently inhibited GAG loss and reversed the reduction in chondrocyte biosynthesis induced by TNFα-treatment of bovine cartilage

**Figure 1:**
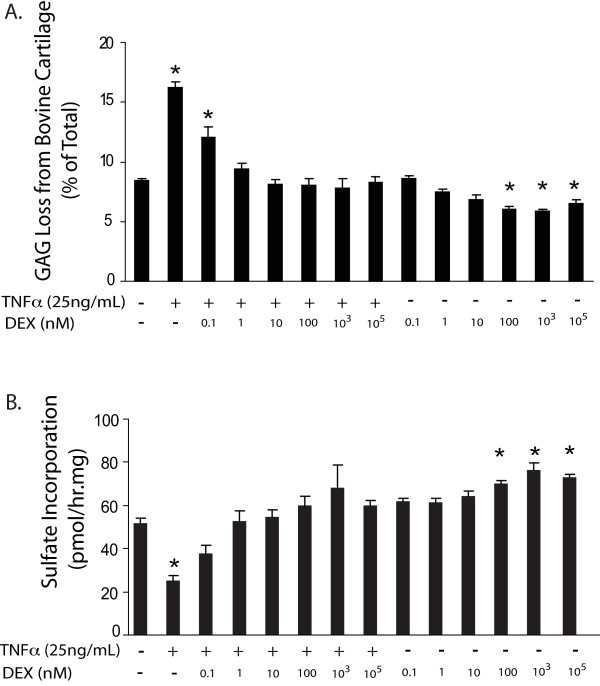
**Dexamethasone dose-respose studies**. **A**) Effect of DEX on TNFα-stimulated GAG loss in bovine cartilage explants. Cartilage tissues were cultured in DEX (0.1 nM-100 μM)-supplemented media, with or without TNFα (25 ng/ml) for six days. The total GAG content of untreated control cartilage was 465.6 ± 23.1 μg GAG/disk (mean ± SEM). DEX, at 1 nM and higher reduced GAG loss induced by TNFα treatment. **B**) Effect of DEX on chondrocyte biosynthetic rates as measured by ^35^S-sulfate incorporation during days 4 to 6. TNFα treatment significantly lowered biosynthesis of sulfated proteoglycans; DEX reversed this inhibition at concentrations of 0.1 nM or higher. Values in A and B are presented as mean ± SEM; *n *= 5 cartilage disks per condition. *= *P *< 0.05 vs. no-treatment control. DEX, dexamethasone; GAG, glycosaminoglycans; SEM, standard error of the mean; TNFα, tumor necrosis factor alpha.

Experiments were performed to test the effect of DEX (0.1 nM to 100 μM) on both TNFα-treated and untreated control cartilage explants (Figure 1). TNFα treatment significantly increased GAG loss to the medium (to 16.2 ± 0.5% of total by six days) compared to that from controls (8.5 ± 0.2%, mean ± SEM), a finding consistent with previous studies [11]. DEX at concentrations of 1 nM or higher reduced GAG loss induced by TNFα treatment to levels that were not significantly different from controls. At concentrations 100 nM and higher, DEX treatment alone suppressed GAG loss to levels below those found in control cultures (Figure 1A).

All cartilage samples from Figure 1A were also radiolabeled with ^35^S-sulfate to measure the rates of proteoglycan biosynthesis in response to treatment conditions. Compared to controls (having ^35^S-sulfate incorporatio*n *= 51.6 ± 2.1 pmol/hour/mg wet weight), TNFα treatment significantly reduced sulfate incorporation to 25.3 ± 2.3 pmol/hour/mg (Figure 1B). In contrast, treatment with TNFα and DEX at concentrations of 0.1 nM and higher showed sulfate incorporation rates, which were not significantly different from controls. Moreover, concentrations of 0.1 μM to 100 μM DEX alone significantly increased sulfate incorporation above control levels (70.0 ± 1.6, 75.9 ± 3.5, and 73.0 ± 1.0 pmol/h/mg, respectively, Figure 1B).

### DEX inhibited GAG loss and biosynthesis reduction in bovine cartilage treated with combinations of mechanical injury, TNFα and IL-6/sIL6R

**Figure 2:**
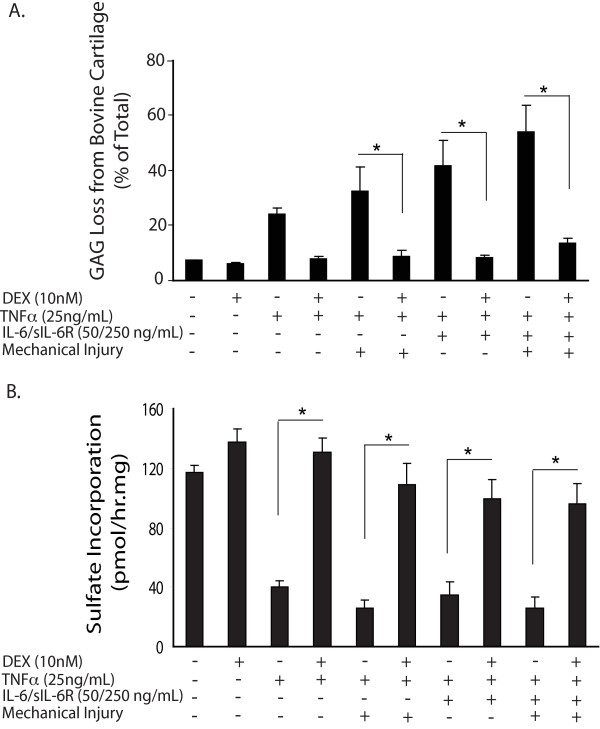
**Effects of Dex on GAG loss and chondrocyte biosynthesis in bovine cartilage treated with combinations of mechanical injury, TNFα and IL-6/sIL-6R**. **A**) Percentage of GAG loss in bovine cartilage in response to six-day treatments. The mean ± SEM total GAG content was 466.3 ± 21.5 μg GAG/disk in the untreated control group. 10 nM DEX significantly reduced GAG loss from conditions involving TNFα plus IL-6/sIL-6R, mechanical injury or both. **B**) Chondrocyte biosynthetic rates measured by ^35^S-sulfate incorporation during days 4 to 6. TNFα, either with or without IL-6/sIL-6R and mechanical injury, significantly lowered biosynthesis of proteoglycan, while the addition of DEX to these conditions blocked the biosynthesis reductions. Values in A and B are presented as mean ± SEM. N = 10 cartilage disks in no-treatment control, DEX, TNFα, and DEX + TNFα conditions. N = 5 cartilage disks in the remaining conditions.* = *P *< 0.05 (only comparisons from selected hypothesis are shown). DEX, dexamethasone; GAG, glycosaminoglycans; IL-6, interleukin-6; SEM, standard error of the mean; sIL-6R, soluble interleukin-6 receptor; TNFα, tumor necrosis factor alpha.

Consistent with our previous findings, TNFα treatment together with mechanical injury or IL-6/sIL-6R or the combination of all three treatments, significantly increased GAG release from bovine cartilage (Figure 2A) [11]. The combined treatment with injury +TNFα IL-6/sIL-6R caused the most severe GAG loss by six days. The addition of 10 nM DEX significantly reduced GAG loss caused by injury +TNFα, TNFα + IL-6/sIL-6R, and injury +TNFα + IL-6/sIL-6R, the latter from 53.6 ± 9.8% down to 13.8 ± 1.5% compared to 7.3 ± 0.2% for controls.

TNFα treatment, either alone or together with mechanical injury, IL-6/sIL-6R or their combination, greatly reduced sulfate incorporation rates (Figure 2B), as seen in our previous study [11]. Importantly, DEX abolished the reduction in biosynthesis caused by all these treatments. For example, treatment with TNFα + IL-6/sIL-6R + injury reduced sulfate incorporation to 26.2 ± 7.2 pmol/hour/mg, whereas the addition of 10 nM DEX to this same condition significantly increased sulfate incorporation to 96.2 ± 13.44 pmol/hour/mg, a level that was not significantly different from no-treatment controls.

### DEX treatment reduced GAG loss in human cartilage explants

**Figure 3:**
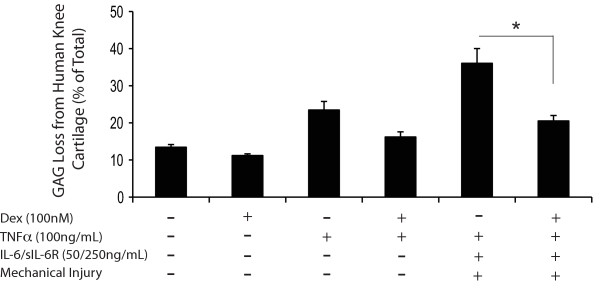
**Effect of DEX on human knee cartilage treated with TNFα and TNFα in combination with injury and IL-6/sIL-6R**. The percentage of GAG loss was measured from 10-day treatments. All cartilage disks included superficial surface. The total GAG content was 168.9 ± 17.1 μg GAG/disk in the untreated control group. 100 nM DEX significantly reduced GAG release induced by treatments with TNFα, IL-6/sIL-6R and mechanical injury. In each condition, *n *= 6 cartilage disks. * = *P *< 0.05 (only comparisons from selected hypothesis are shown). DEX, dexamethasone; GAG, glycosaminoglycans; IL-6, interleukin-6; sIL-6R, soluble interleukin-6 receptor; TNFα, tumor necrosis factor alpha.

Treatment with injury + TNFα + IL-6/sIL-6R greatly increased GAG loss from human knee cartilage (to 36.0 ± 4% of total, Figure 3), consistent with our previous report [11]. Under these conditions, the addition of 100 nM DEX significantly reduced GAG loss to 20.5 ± 1.5%, but showed no effect on sulfate incorporation (data not shown).

### Pre-treatment with DEX reduced GAG loss and increased sulfate incorporation in TNFα-treated cartilage

**Figure 4:**
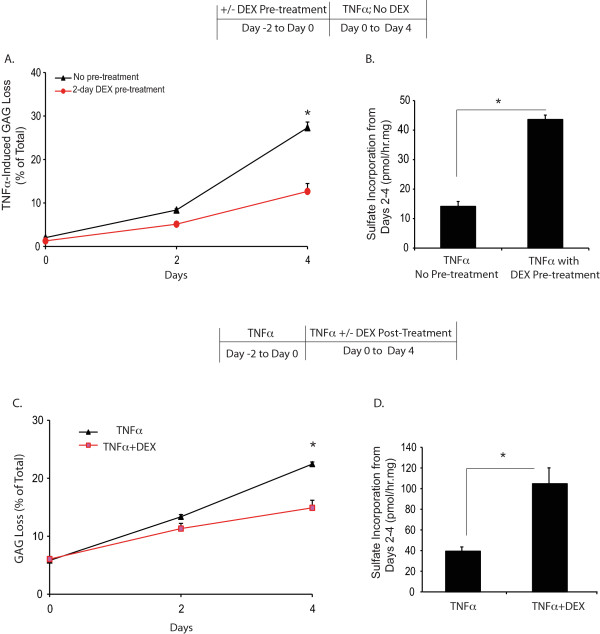
**DEX Pre and post treatment**. **A**, Cumulative GAG loss and **B**, sulfate incorporation (measured in the last two days) from bovine cartilage samples pre-treated with 10 nM DEX for two days prior to a four-day TNFα treatment. On Day 4, Cartilage samples pre-incubated with DEX released significantly less GAG, and showed significantly higher proteoglycan synthesis in the TNFα treatment compared to samples without DEX pre-treatment. **C**, Cumulative GAG loss and **D**, sulfate incorporation (measured in the last two days) in bovine cartilage samples treated with TNFα, in the presence or absence of 10 nM DEX, with a two-day pre-exposure to TNFα. DEX treatment introduced after the TNFα pre-treatment showed significantly reduced GAG loss and increased sulfate incorporation on Day 4. In each condition, *n *= 5 cartilage disks. * = *P *< 0.05 (only comparing the GAG loss difference between conditions on Day 4). DEX: dexamethasone; GAG: glycosaminoglycans; TNFα: tumour necrosis factor alpha.

Bovine cartilage samples were pre-incubated with 10 nM DEX for two days and then cultured in medium containing TNFα, without DEX, for an additional four days. The pre-treatment with DEX significantly reduced TNFα-induced GAG loss by Day 4 (Figure 4A), and significantly increased the sulfate incorporation rate compared to the condition without DEX pre-treatment (Figure 4B).

### Post-treatment with DEX reduced GAG loss and increased sulfate incorporation in TNFα-treated cartilage

We next examined whether DEX would exert anti-catabolic effects in cartilage samples where matrix degradation had already been induced by cytokine stimulation. All cartilage samples were pre-incubated with TNFα for two days (starting at Day -2 in Figure 4C). Afterwards, starting at Day 0, one group of samples was cultured in medium with TNFα +10 nM DEX, while a second group was treated with TNFα alone. After the two-day pre-incubation with TNFα, disks from both groups had lost approximately 6% of total GAG (Day 0, Figure 4C). The addition of DEX significantly attenuated GAG loss and increased proteoglycan biosynthesis by Day 4 (Figure 4C, D).

### The anti-catabolic effects of DEX were glucocorticoid receptor (GR) mediated

**Figure 5:**
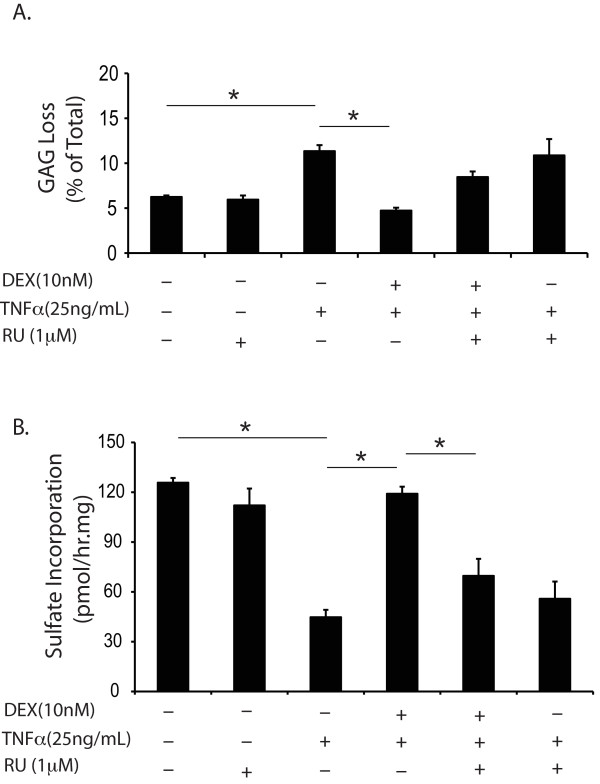
**The percentage of GAG loss in six days (A) and proteoglycan biosynthesis measured from days 4 to 6 in bovine cartilage in response to TNFα, DEX and glucocorticoid receptor antagonist, RU 486 (B)**. RU reversed the effect of DEX in sulfate incorporation. In each condition, *n *= 5 cartilage disks. * = *P *< 0.05 (only selected comparisons are shown). DEX, dexamethasone; GAG, glycosaminoglycans; RU486, a glucocorticoid receptor antagonist; TNFα, tumor necrosis factor alpha.

To assess whether the inhibition of GAG loss and the increase in proteoglycan biosynthesis in DEX-treated cartilage were GR mediated, bovine explants were treated with the GR antagonist RU486 in addition to TNFα ± DEX. As shown in Figure 5, TNFα significantly increased GAG loss and reduced sulfate incorporation rate; the addition of DEX significantly reduced the release of GAGs and increased the sulfate incorporation rate. The effects of DEX on biosynthesis were significantly reversed by the presence of RU486, though the increase in GAG release upon addition of RU486 was not statistically significant. RU486 alone had no effect on either normal controls or TNFα-treated samples.

### Effects of DEX, TNFα and mechanical injury on chondrocyte gene expression

**Figure 6:**
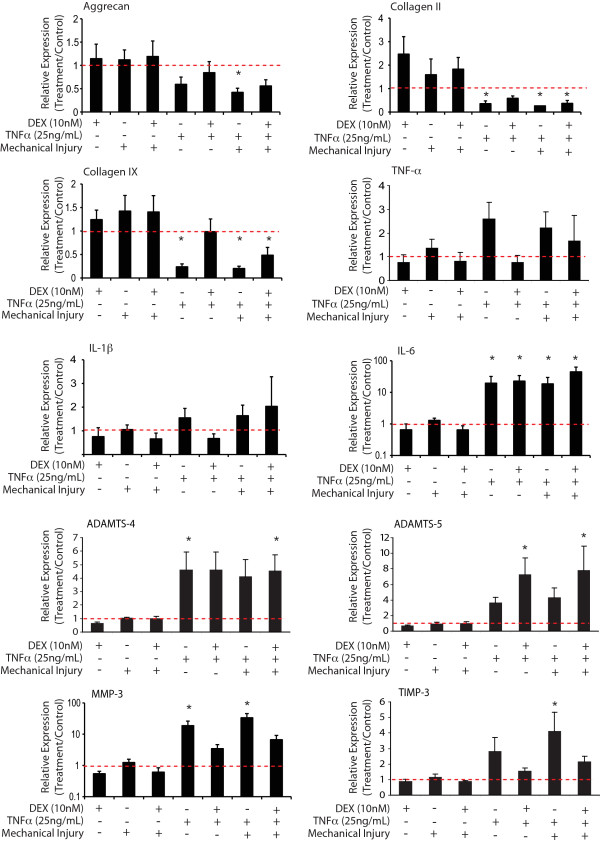
**Changes in chondrocyte gene expression after four-day treatments with DEX, TNFα, injury and their combinations**. Values at the y-axis represent the expression level of each gene normalized to that of the no-treatment control (y = 1, dotted line). Values are mean ± SEM, *n *= 6 animals. * = *P *< 0.05 vs. no-treatment control. DEX: dexamethasone; SEM: standard error of the mean; TNFα: tumor necrosis factor alpha.

Real-time qPCR was performed to determine bovine chondrocyte gene expression responses to four-day treatments with DEX, TNFα and mechanical injury alone and in combinations (Figure 6). Matrix molecules collagen II and IX responded to both TNFα and TNFα + injury treatments with a significant decrease in mRNA levels. DEX treatment increased the expression of both genes in cartilage treated with TNFα to levels not significantly different than controls. Aggrecan core protein mRNA levels were significantly decreased in response to TNFα + injury; however, the addition of DEX resulted in mRNA levels not significantly different than controls. IL-6 mRNA levels were increased significantly by treatments involving TNFα, regardless of the presence of DEX or mechanical injury treatment. Treatment conditions had no significant effect on TNFα or IL-1β mRNA levels.

Analysis also showed that TNFα alone significantly up-regulated the levels of ADAMTS-4 and MMP-3 mRNA. TNFα + injury significantly increased ADAMTS-4 and ADAMTS-5 mRNA levels in the presence of DEX. Additional genes, related to protease and protease inhibition, were up-regulated in response to the TNFα + injury treatment, including TIMP-3 and MMP-3. Among the matrix proteases, only MMP-3 mRNA showed reduced expression in response to DEX + TNFα and DEX + TNFα+ injury treatments, whereas ADAMTS-5 mRNA levels were not down-regulated in the presence of DEX.

### iNOS and nitrite

**Figure 7:**
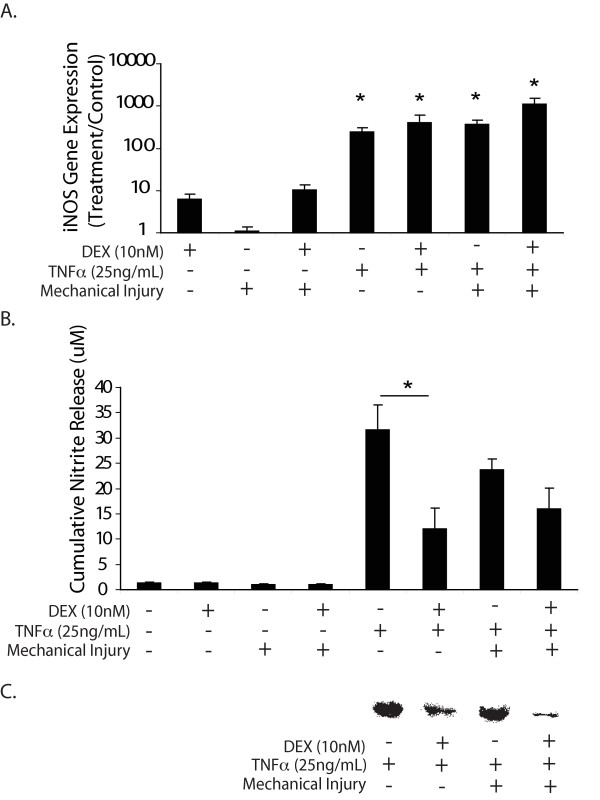
**Effects of DEX on iNOS gene and protein expression and nitrite production**. **A**. iNOS relative gene expression. Values at the y-axis represent the expression level of each gene normalized to that of the no-treatment control (y = 1). Values are mean ± SEM, *n *= 6 animals. * = *P *< 0.05 vs. no-treatment control. **B**. Nitrite released to the medium from days 0 to 4. In each condition, *n *= 6 cartilage disks. *= *P *< 0.05 (only comparisons from selected hypotheses are shown). **C**. Western blot for iNOS protein. DEX, dexamethasone; iNOS, inducible nitric oxide synthase; SEM, standard error of the mean.

iNOS message expression was significantly elevated in response to all treatments with TNFα, but not by injury alone. The induction of iNOS mRNA was not abrogated by the addition of DEX (Figure 7A). However, DEX significantly reduced the amount of nitrite released to the conditioned medium caused by TNFα treatment alone (Figure 7B). Western blot analysis showed that iNOS protein levels in the TNFα + DEX and TNFα + injury + DEX conditions were markedly reduced compared to these same conditions without DEX (Figure 7C).

### Proprotein convertase (PC) inhibitor decreased GAG loss induced by cytokine and mechanical injury treatments

**Figure 8:**
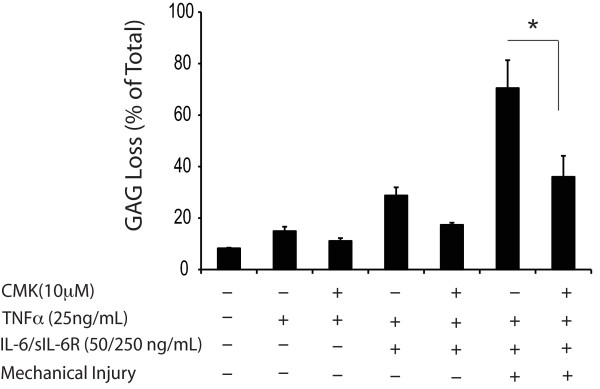
**Effects of CMK on GAG loss in treatment conditions**. Percentage of GAG loss from bovine cartilage in response to treatments with TNFα, IL-6/sIL-6R, mechanical injury and CMK, a general inhibitor for PCs. CMK significantly reduced GAG loss caused by TNFα + IL-6 + mechanical injury. In each condition, *n *= 5 cartilage disks. *P < 0.05 (only comparisons from selected hypotheses are shown). CMK, decanoyl-RVKR-CMK, a general proprotein convertase inhibitor; GAG, glycosaminoglycans; IL-6, interleukin 6; PC, proprotein convertase; sIL-6R, soluble interleukin-6 receptor; TNFα, tumor necrosis factor alpha.

To assess the role of PC in cartilage degradation, a general PC inhibitor, decanoyl-RVKR-CMK, was added to the conditions of TNFα, TNFα + IL-6/sIL-6R, and TNFα + IL-6/sIL-6R + injury. 10 μM CMK significantly decreased GAG release induced by TNFα + IL-6/sIL-6R + injury (Figure 8).

## Discussion

The objective of this study was to determine the effects of DEX on cartilage proteoglycan degradation and synthesis in response to combined treatments with mechanical injury and pro-inflammatory cytokines. We previously reported that co-stimulation of cartilage with TNFα and IL-6/sIL-6R caused significantly more GAG release than either cytokine alone, in both immature bovine knee and adult human knee and ankle cartilage [11]. Moreover, mechanical injury substantially potentiated the combined catabolic effects of TNFα and IL-6/sIL-6R by inducing severe matrix degradation. In this study, we first demonstrated that DEX, over a wide range of concentrations (1 nM to 100 μM), completely blocked TNFα-induced GAG loss and reversed the reduction in biosynthesis caused by TNFα in bovine cartilage (Figure 1). Even in the absence of cytokine stimulation, cartilage disks exposed to higher concentrations (that is, 0.1 to 100 μM) of DEX released fewer GAGs and showed increased sulfate incorporation compared to control samples.

Importantly, DEX (10 nM) also restored proteoglycan biosynthesis and inhibited GAG loss caused by the treatments with TNFα + IL-6/sIL-6R, injury + TNFα, and injury + TNFα + IL-6/sIL-6R (Figures 2 and 3). The proteoglycan fragments produced under these conditions were previously found to be generated by aggrecanases, not MMPs [11]. Thus, the inhibitory effect of DEX on matrix degradation may involve modulating the proteolytic activities of aggrecanases. Recently, Malfait *et al. *demonstrated that DEX blocked aggrecanase activity in an *in vivo *model of cartilage degradation: intra-articular injection of TNFα in rats resulted in aggrecanase-generated proteoglycan degradation, which could be inhibited by either an aggrecanase inhibitor or DEX, but not a non-steroidal anti-inflammatory drug [44].

Surprisingly, DEX did not abrogate GAG release via a substantial reduction in aggrecanase transcriptional levels. In particular, the mRNA levels of ADAMTS-4 and -5 in response to TNFα + injury treatment remained elevated in the presence of DEX (Figure 6). Similarly, DEX did not down-regulate the gene expression of iNOS, although it markedly reduced the level of iNOS protein as well as nitric oxide production in both cytokine-stimulated and cytokine plus injury-treated cartilage (Figure 7). Previous studies by Guerne *et al. *[45] and Shalom-Barak *et al. *[46] also reported the down-regulation of cytokine-induced nitric oxide synthesis in human chondrocytes by glucocorticoids. Therefore, DEX may not regulate matrix degradation at the transcriptional level alone. Aggrecanase activity can be affected at multiple levels, including altered protein expression, pro-enzyme activation and binding to aggrecan via the C-terminal thrombospondin motif. In this study, we hypothesized that DEX may block aggrecanase activity by inhibiting the activation of latent pro-ADAMTS-4 and -5. We showed that blocking PC activity significantly reduced GAG loss in the cytokine plus injury treatments (Figure 8), consistent with the important role of PCs in proteoglycan degradation. Others have made similar observations with TNFα-treated cartilage [26]. Ongoing studies focus on how DEX modulates PC activities as well as other possible mechanisms involved in DEX-induced inhibition of proteoglycan degradation.

We further demonstrated that treating cartilage with DEX either before or after TNFα stimulation significantly reduced GAG loss and increased proteoglycan biosynthesis (Figure 4). These observations suggest that the effects of DEX are long lasting and may provide protection against further exposure to cytokines. Even when catabolic processes have already begun in cartilage, DEX treatment could still suppress GAG loss and increase biosynthesis.

In this study, we also observed that DEX (100 nM) significantly reduced GAG loss in human cartilage (though no stimulation of proteoglycan biosynthesis was seen). Hardy et al. also observed that DEX blocked IL-1 stimulated proteoglycan degradation in OA cartilage cultured with synovium [47]. Guerne *et al. *reported that DEX inhibited the down-regulating effect of IL-1 and IL-6/sIL-6R on proteoglycan synthesis, enhancing matrix synthesis in normal, and, to a lesser extent in osteoarthritic human chondrocytes [45]. Together these reports indicate DEX may also produce favorable responses in human cartilage.

GCs have been widely used in the treatment of joint diseases [12,13]. Most studies and trials reported beneficial responses, including significantly greater reduction of pain and tenderness, and increased motion in the injected joint [48,49]. However, because the mechanism of GCs in cartilage function is not well understood, and since there have been anecdotal reports of GC-related side effects when treating joint diseases, the chronic use of GCs in OA treatment remains controversial. It has been noted that the reports describing negative effects of GCs often involved either frequent injections or high dosages [50]. More careful reviews have shown that the efficacy of GC is dependent on the concentration used [51]. In order to avoid complications, longer intervals between GCs injections for the weight-bearing joints have been recommended [52]. Future intra-articular treatments may also involve the use of micro and nano drug delivery technologies, which could enable local, controlled release of GC and avoid the problems associated with frequent injections and over dosage [53]. There have not been any reports on the long-term effects of GC treatment on joint injury. However, the current study suggests the concept that immediate treatment of DEX in the injured knee may greatly retard the initial progression of cartilage degradation. Moreover, our data suggest that even delayed administration of DEX may also be beneficial, thereby providing the clinician with a window of therapeutic opportunity.

## Conclusions

Acute knee injury initiates cascades of catabolic events in joint tissues, including mechanical disruption of cartilage matrix and increasing synovial fluid concentrations of pro-inflammatory cytokines. Glucocorticoid treatment of cartilage can effectively abolish matrix degradation induced by the combination of pro-inflammatory cytokines and injury. We suggest that DEX can protect cartilage matrix from post-traumatic degenerative changes by both suppressing catabolic activities and maintaining matrix biosynthesis. DEX inhibits catabolism of proteoglycans by modulating aggrecanase proteolytic activities within cartilage. DEX, therefore, may be a promising therapeutic agent for preventing cartilage degeneration and post-traumatic osteoarthritis in individuals following joint injury.

## Abbreviations

ACL: anterior cruciate ligament; ADAMTS: a disintegrin and metalloproteinase with thrombospondin motifs; CMK: decanoyl-RVKR-CMK, a general proprotein convertase inhibitor; DEX: dexamethasone; DMEM: Dulbecco's Modified Eagle's Medium; DMMB: dimethylmethylene blue; GAG: glycosaminoglycans; GC: glucocorticoid; GR: glucocorticoid receptor; HEPES: 4-(2-hydroxyethyl)-1-piperazine ethane sulfonic acid; IL-6: interleukin-6; iNOS: inducible nitric oxide synthase; MMP-3: matrix metalloproteinase-3; OA: osteoarthritis; PACE: furin/paired basic amino-acid cleaving enzyme; PC: proprotein convertase; qPCR: quantitative real time polymerase chain reaction; RA: rheumatoid arthritis; rhTNFα: recombinant human tumor necrosis factor alpha; RU486: a glucocorticoid receptor antagonist; SEM: standard error of the mean; sGAG: sulfated glycosaminoglycans; sIL-6R: soluble interleukin-6 receptor; TIMP-3: tissue inhibitor of metalloproteinase-3; TNFα: tumor necrosis factor alpha.

## Competing interests

The authors declare that they have no competing interests.

## Authors' contributions

YCL conducted all the experiments, analyzed the data, conceived of and designed the studies, confirmed data analysis and wrote the manuscript. CE and AJG conceived of and designed the studies, confirmed data analysis and wrote the manuscript. All authors read and approve the final manuscript.
